#  Incidência de depressão e fatores associados em idosos de Bagé, Rio Grande do Sul, Brasil 

**DOI:** 10.1590/0102-311XPT248622

**Published:** 2023-11-13

**Authors:** Pâmela Moraes Volz, Alitéia Santiago Dilélio, Luiz Augusto Facchini, Lenice de Castro Muniz de Quadros, Elaine Tomasi, Marciane Kessler, Louriele Soares Wachs, Karla Pereira Machado, Mariângela Ulhmann Soares, Elaine Thumé

**Affiliations:** 1 Universidade Federal de Pelotas, Pelotas, Brasil.; 2 Universidade Federal do Rio Grande, Rio Grande, Brasil.

**Keywords:** Incidência, Saúde do Idoso, Depressão, Estudos Longitudinais, Incidence, Health of the Elderly, Depression, Longitudinal Studies, Incidencia, Salud del Anciano, Depresión, Estudios Longitudinales

## Abstract

Com o objetivo de avaliar a incidência cumulativa de depressão e seus fatores associados na população idosa, residente na zona urbana do Município de Bagé, Rio Grande do Sul, Brasil, realizou-se um estudo de coorte, prospectivo, entre 2008 e 2016/2017. A análise foi restrita a 615 idosos com informações completas na *Escala de Depressão Geriátrica* (GDS-15), tanto na linha de base como no seguimento, que não apresentavam depressão no ano de 2008. Para calcular as razões de incidência bruta e ajustadas e intervalo de 95% de confiança, foi utilizada a regressão de Poisson com ajuste robusto de variância, incluindo as variáveis da linha de base. Empregou-se um modelo hierárquico de quatro níveis de determinação. As variáveis foram controladas para aquelas do mesmo nível ou dos níveis superiores, sendo estabelecido o valor de p ≤ 0,20 para permanecer no modelo de análise. Observou-se que, em 2008, 523 idosos não tinham depressão e 92 haviam sido diagnosticados com a doença. Em 2016/2017, dos 523 indivíduos sem depressão na medida de linha de base, 10,3% apresentaram resultado positivo no rastreamento (casos incidentes), enquanto 89,7% dos idosos permaneceram livres do problema. Dos 92 idosos com depressão em 2008, 32,6% continuaram referindo a sintomatologia depressiva no acompanhamento e 67,3% apresentaram remissão dos sintomas. Sair de casa uma ou nenhuma vez e apresentar incapacidades para o desenvolvimento de atividades funcionais e instrumentais da vida diária se associaram com maior risco de apresentar rastreamento positivo para depressão. Os resultados reforçam o caráter multidimensional e dinâmico da depressão, que alterna episódios curtos e longos, podendo se tornar recorrente e de curso crônico.

## Abstract (en)

To evaluate the cumulative incidence of depression and its associated factors in the older population living in the urban area of the municipality of Bagé, Rio Grande do Sul State, Brazil, a prospective cohort study was conducted from 2008 to 2016/2017. The analysis was restricted to 615 older adults with complete information on the *Geriatric Depression Scale* (GDS-15), both at baseline and at follow-up, and who did not present depression in 2008. To calculate crude and adjusted incidence ratios and 95% confidence interval, Poisson regression with robust variance adjustment was used, including the baseline variables. A hierarchical model of four levels of determination was used. The variables were controlled for those of the same or of higher levels, and the p-value ≤ 0.20 was established to remain in the analysis model. In 2008, 523 older people did not have depression and 92 had been screened with the disease. In 2016/2017, of the 523 individuals without depression in the baseline measure, 10.3% tested positive at screening (incident cases), whereas 89.7% of the older adults remained free of the condition. Of the 92 older adults with depression in 2008, 32.6% continued to present depressive symptoms at follow-up and 67.3% had remission of symptoms. Leaving home once or never and being incapable of doing functional and instrumental activities of daily living were associated with a higher risk of positive screening for depression. The results reinforce the multidimensional and dynamic character of depression, which alternates short and long episodes, and may become recurrent and chronic.

## Resumen

Con el objetivo de evaluar la incidencia acumulada de la depresión y sus factores asociados en la población anciana que vive en el área urbana de la ciudad de Bagé, Rio Grande do Sul, Brasil, se realizó un estudio de cohorte, prospectivo, en el período entre 2008 y 2016/2017. Se analizaron a 615 ancianos que tenían información completada en la *Escala de Depresión Geriátrica* (GDS-15), tanto en la línea de base como en el seguimiento, y aquellos que no tuvieron depresión en 2008. Para calcular los índices de incidencia brutos y ajustados y el intervalo de 95% confianza, se utilizó la regresión de Poisson con un ajuste robusto de la varianza, incluidas las variables de la línea de base. Se empleó un modelo jerárquico de cuatro niveles de determinación. Las variables se controlaron por aquellas del mismo nivel o niveles superiores, y se estableció un valor de p ≤ 0,20 para permanecer en el modelo de análisis. Se observó que 523 ancianos en 2008 no tuvieron depresión y 92 recibieron diagnóstico de la enfermedad. Para el 2016-2017, de los 523 individuos sin depresión en la medida de línea de base, el 10,3% de ellos se detectó con la enfermedad en el cribado (casos incidentes), mientras que el 89,7% no la manifestó. De los 92 ancianos con depresión en 2008, el 32,6% informaron tener síntomas depresivos en el seguimiento y el 67,3% presentó remisión de los síntomas. Salir de casa una sola vez o ninguna y sentir incapaz de realizar las actividades funcionales e instrumentales de la vida diaria estuvieron asociadas a un mayor riesgo de tener cribado positivo para la depresión. Los resultados evidenciaron el carácter multidimensional y dinámico de la depresión, que oscila entre episodios cortos y largos, y puede llegar a ser recurrente y crónica.

## Introdução

A depressão é um relevante problema de saúde pública, sendo a principal causa de incapacidade física e mental no mundo e uma das principais contribuintes para a carga global de doenças ^1^. Entre os idosos, caracteriza-se como o transtorno mental mais frequente, associado a elevado grau de sofrimento psíquico, maior risco de morbidade e mortalidade, negligência no autocuidado, maior utilização de serviços de saúde, menor adesão ao tratamento medicamentoso e a regimes terapêuticos e redução da qualidade de vida ^2^^,^^3^^,^^4^^,^^5^.

De acordo com a Organização Mundial da Saúde (OMS), a depressão atinge 4,8% da população mundial, acometendo 5,8% (11,5 milhões) dos brasileiros ^6^. Munhoz et al. ^7^, com informações provenientes da *Pesquisa Nacional de Saúde* (PNS) de 2013 e utilizando o *Questionário de Saúde do Paciente* (PHQ-9 - *Patient Health Questionnaire-9*), identificaram aumento significativo da prevalência de depressão em função da idade. O estudo encontrou maior razão de prevalência (RP) entre os grupos populacionais com idades entre 40-49 anos (RP = 1,36; intervalo de 95% de confiança - IC95%: 1,08-1,69), 50-59 anos (RP = 1,56; IC95%: 1,24-1,97) e 80 anos ou mais (RP = 1,63; IC95%: 1,11-2,40) em comparação ao grupo de referência (18-29 anos) ^7^.

Os determinantes da incidência de depressão em idosos incluem aspectos sociais, comportamentais, culturais, ambientais, econômicos, políticos, familiares e de saúde. Estudos de coorte desenvolvidos por Gureje et al. ^8^ e por Luppa et al. ^9^ encontraram, respectivamente, 1,63 (IC95%: 1,30-2,06) e 2,93 vezes (IC95%: 1,50-5,73) mais chances de depressão em mulheres, em comparação com os homens. Estudo desenvolvido por Pálsson et al. ^10^ identificou que, entre 70 e 85 anos, a incidência de depressão foi de 12/1.000 pessoas-ano entre os homens e de 30/1.000 pessoas-ano entre as mulheres. Forsell & Winblad ^11^ verificaram que idosos que não receberam visitas ao longo do mês em que foram entrevistados e que não tinham amigos e nem conhecidos para conversar livremente tiveram mais chances de desenvolver depressão do que os idosos que não relataram essas condições (*odds ratio* - OR = 3,30; IC95%: 1,20-5,40). Bojorquez-Chapela et al. ^12^ identificaram que idosos funcionalmente independentes são menos propensos a desenvolver depressão (OR = 0,4; IC95%: 0,27-0,79), em comparação a idosos com incapacidade funcional. Para Collard et al. ^13^, idosos com fragilidade física tiveram 1,26 vez mais chance de apresentar depressão (IC95%: 1,09-1,45), se comparados com aqueles sem fragilidade. Estudos desenvolvidos por Luppa et al. ^9^, Nascimento et al. ^14^, Michikawa et al. ^15^, Saito et al. ^16^, Kim et al. ^17^ e Mast et al. ^18^ demonstraram que presença de dor articular crônica, problemas de audição, diabetes, cardiopatias, doenças cerebrovasculares, baixa densidade lipoproteica, síndrome metabólica, doenças vasculares ou problemas cognitivos estão relacionados à maior incidência de depressão.

No Brasil, pouco se sabe sobre a incidência de depressão entre a população idosa, particularmente de seus determinantes e de suas consequências para a saúde. Foram encontrados dois estudos brasileiros longitudinais e de base populacional que abordaram essa temática ^14^^,^^19^. O estudo dos processos do envelhecimento saudável de Juiz de Fora, Minas Gerais, realizado com idosos de 60 anos ou mais, encontrou uma incidência cumulativa de depressão de 15,2%, após três anos de acompanhamento ^14^. O estudo de coorte de idosos de Bambuí, Minas Gerais, desenvolvido com indivíduos de 60 anos ou mais, encontrou uma densidade de incidência de 46/1.000 pessoas-ano, após 10 anos de seguimento ^19^. Pesquisas recentes sinalizam os sintomas depressivos como preditores de fragilidade, incapacidade e mortalidade na população idosa ^20^^,^^21^. Análises de incidência são fundamentais para subsidiar políticas de fomento a prevenção, diagnóstico, tratamento e atenção integral à depressão, reduzindo a incapacidade e a mortalidade prematura em idosos. Para contribuir com o conhecimento sobre o problema, este estudo de coorte avaliou a incidência cumulativa de depressão e os fatores associados na população idosa após nove anos de acompanhamento.

## Material e métodos

Estudo de coorte, prospectivo, de base populacional, intitulado *Saúde do Idoso Gaúcho de Bagé* (SIGA-Bagé), realizado com idosos de 60 anos ou mais, residentes na área de abrangência dos serviços de atenção básica à saúde da zona urbana do município.

Bagé localiza-se no extremo sul do Brasil, tinha uma população estimada em 120.943 habitantes em 2018 e densidade demográfica de 28,52 habitantes/km^2^. O município está situado a pouco mais de 350km de Porto Alegre (capital do estado), faz fronteira com o Uruguai ao sul e sua principal base econômica é a atividade agropecuária. O Índice de Desenvolvimento Humano Municipal (IDH-M) em 2010 era de 0,740 e o produto interno bruto (PIB) era de R$ 21.930,00 *per capita*/ano em 2016. A escolha do município como local de estudo em 2008 considerou a taxa de cobertura da Estratégia Saúde da Família (ESF) (51%), que era a maior entre os municípios com mais de 100 mil habitantes do Estado do Rio Grande do Sul. Além disso, a proporção de indivíduos com 60 anos ou mais (12%) era superior em comparação ao país (10%) ^22^.

No estudo de linha de base (ELB) em 2008, a amostra de 1.713 idosos foi localizada por busca sistemática nas áreas de abrangência das 20 unidades básicas de saúde (UBS) da zona urbana. Todos os moradores com 60 anos ou mais residentes nos domicílios selecionados foram convidados a participar, dos quais 1.593 (93%) foram entrevistados. Os idosos foram selecionados, sorteando diferentes pontos de partida com pulo sistemático de cinco domicílios entre as residências, a fim de garantir a equiprobabilidade e a distribuição da amostra no território, estimando-se a presença de um idoso a cada três domicílios. Os dados foram coletados por meio de um questionário estruturado com perguntas pré-codificadas após um estudo piloto. Informações detalhadas sobre a metodologia do ELB foram descritas por Thumé et al. ^23^. O acompanhamento foi realizado no período de setembro de 2016 a agosto de 2017. Tanto no ELB quanto no acompanhamento, a coleta de dados foi realizada no domicílio do idoso por entrevistadores capacitados. O controle de qualidade foi feito com a repetição parcial de 10% das entrevistas, contendo perguntas-chave para avaliar a confiabilidade do instrumento.

Para o desfecho, foi utilizada a *Escala de Depressão Geriátrica* (GDS-15 - *Geriatric Depression Scale*) em 2008 e 2016/2017, na versão reduzida de Yesavage et al. ^24^, com 15 perguntas válidas para a avaliação e o rastreio de sintomas depressivos em idosos. Com respostas dicotômicas (sim; não), a escala aborda o sentimento do idoso na maioria dos 30 dias anteriores à entrevista. O escore ≥ 6 indica um rastreamento positivo, tendo produzido índices de sensibilidade de 85,4% e especificidade de 73,9% para o diagnóstico de episódio depressivo de acordo com a Classificação Internacional de Doenças, 10ª revisão (CID-10) ^25^. Para a análise de incidência cumulativa, restringiu-se a amostra a 615 idosos com informações completas na escala GDS-15, nos dois acompanhamentos.

As variáveis independentes foram obtidas em 2008 e compreenderam as seguintes características: (a) sociodemográficas: sexo (masculino; feminino), idade (< 75 anos; 75 anos ou mais), cor da pele (branca; preta/parda/amarela/indígena), situação conjugal (casado ou com companheiro; solteiro ou separado ou viúvo), anos completos de estudo (8 anos ou mais; 1-7 anos; nenhum), classificação econômica pela Associação Brasileira de Empresas de Pesquisas (ABEP) 2018 (A-B; C; D-E), trabalho no último mês (sim; não) e aposentadoria (sim; não); (b) comportamentais: saiu de casa nos últimos 30 dias (não saiu nenhum dia; saiu 1 vez por semana; saiu 2 vezes por semana ou mais), tabagismo (fumante/ex-fumante; não fumante) e consumo de bebida alcoólica nos últimos 30 dias (não; sim); (c) rede de apoio social (escala *Estudo Longitudinal Dinamarquês sobre o Comportamento na Saúde*, do inglês *Danish Longitudinal Health Behavior Study*) ^26^ (apoio moderado e forte - 10 pontos ou mais; apoio fraco - 5 a 9 pontos); e (d) número de morbidades com diagnóstico médico: hipertensão arterial sistêmica, diabetes mellitus, problemas cardíacos, problema pulmonar, artrite e artrose, problemas de visão e problemas de audição (nenhuma; 1; 2 ou mais). A incapacidade foi avaliada pelo desempenho e pela independência do idoso (0: sem incapacidade; 1 e 2: com incapacidade) a partir das escalas de Lawton ^27^ (sem incapacidade: 5 pontos; com incapacidade: 0 a 4 pontos) e Katz ^27^ (sem incapacidade: entre 25 e 27 pontos; com incapacidade: de 0 a 24 pontos).

Para a análise descritiva, foram calculados as incidências e seus respectivos IC95%. Os riscos relativos (RR), expressão das razões de incidência, brutos e ajustados e o IC95% foram calculados por meio da regressão de Poisson ^28^ com ajuste robusto de variância, incluindo as variáveis da linha de base. Empregou-se um modelo hierárquico de quatro níveis de determinação da saúde, proposto por Dahlgren & Whitehead ^29^. O primeiro foi composto pelas variáveis sociodemográficas; o segundo, pelas variáveis comportamentais e rede de apoio social. O terceiro nível contemplou o número de morbidades com diagnóstico médico e o quarto incluiu as atividades básicas e instrumentais da vida diária. As variáveis foram controladas para aquelas do mesmo nível ou dos níveis superiores, sendo estabelecido o valor de p ≤ 0,20 para permanecer no modelo de análise. Foi considerado o valor de p do teste de Wald de heterogeneidade e de tendência linear de < 0,05 para as associações significativas. Os dados foram analisados no programa estatístico Stata, versão 12.0 (https://www.stata.com).

O projeto foi aprovado pelo Comitê de Ética em Pesquisa da Faculdade de Medicina da Universidade Federal de Pelotas, em 29 de maio de 2014 (parecer nº 678.664), seguindo os preceitos da *Resolução nº 466/2012*. Os princípios éticos foram assegurados a partir da assinatura do Termo de Consentimento Livre e Esclarecido pelos participantes.

## Resultados

No ELB, foram entrevistados 1.593 idosos (proporção de resposta de 93%). No acompanhamento realizado em 2016/2017, 1.314 idosos (82,5%) foram localizados, dos quais 735 (55,9%) foram entrevistados novamente e 579 (44,1%) foram a óbito, confirmado pelo Sistema de Informação sobre Mortalidade (SIM), até agosto de 2017. Entre os 279 (17,5%) indivíduos que não foram acompanhados, 81 (29%) foram recusas e 198 (71%) foram perdas, principalmente por não localização do endereço, mudança para outros municípios e institucionalização.

Nas duas avaliações, houve predomínio de indivíduos do sexo feminino, de cor branca, com 1-7 anos de escolaridade, que não trabalharam no último mês, aposentados, que saíram de casa duas vezes por semana ou mais, fumante/ex-fumante e que não consumiam bebida alcoólica, com diagnóstico médico de duas ou mais morbidades, sem incapacidade para a realização de atividades básicas e instrumentais da vida diária (Tabela 1).

**Tabela 1:** Distribuição da amostra na linha de base e no acompanhamento. Bagé, Rio Grande do Sul, Brasil, 2008 (n = 1.593) e 2016/2017 (n = 735).

Variável	2008		2016/2017		Valor de p
	n	%	n	%	
Sexo					0,471
Masculino	593	37,2	254	34,6	
Feminino	1.000	62,8	481	65,4	
Idade (anos)					0,000
< 75	1.096	69,0	309	42,0	
75 ou mais	497	31,0	426	58,0	
Cor					0,070
Branca	1.252	78,6	604	82,2	
Preta/Parda/Amarela/Indígena	341	21,4	131	17,8	
Situação conjugal					0,007
Casado ou com companheiro	816	51,2	310	42,2	
Solteiro, separado ou viúvo	776	48,8	425	57,8	
Escolaridade (anos completos de estudo)					0,858
8 ou mais	342	21,5	162	22,2	
1-7	868	54,5	398	54,6	
Nenhum	382	24,0	169	23,2	
Classificação econômica (ABEP)					0,007
A/B	429	27,1	105	14,6	
C	615	39,0	283	39,3	
D/E	537	34,0	332	46,1	
Trabalho no último mês					0,000
Sim	200	12,6	67	9,1	
Não	1.392	87,4	667	90,9	
Aposentadoria					0,003
Sim	1.142	71,7	584	79,7	
Não	451	28,3	149	20,3	
Saiu de casa no último mês					0,188
2 vezes por semana ou mais	1.004	63,0	481	66,5	
1 ou nenhuma vez por semana	589	37,0	242	33,5	
Tabagismo					0,114
Fumante/Ex-fumante	875	55,0	365	50,1	
Não fumante	717	45,0	363	49,9	
Consumo de bebida alcoólica (último mês)					0,362
Não	1.329	84,0	623	85,6	
Sim	255	16,1	105	14,4	
Número de morbidades com diagnóstico médico					0,001
1	276	18,6	224	30,8	
2 ou mais	1.200	81,3	504	69,2	
Incapacidade (AVD e AIVD)					0,098
Sem incapacidade	1.423	89,6	622	87,1	
Com incapacidade	166	10,4	92	13,0	

ABEP: Associação Brasileira de Empresas de Pesquisa; AIVD: atividades instrumentais de vida diária; AVD: atividades de vida diária.

No acompanhamento, observou-se mudança significativa na proporção da categoria predominante em relação ao ELB para as variáveis idade, situação conjugal e classe econômica. O predomínio de indivíduos com menos de 75 anos deu lugar ao de idosos com 75 anos ou mais; o de casados e com companheiro, ao de solteiros, separados ou viúvos; o de idosos de classe econômica C, ao de pertencentes às classes D/E (Tabela 1).

**Figura 1:**
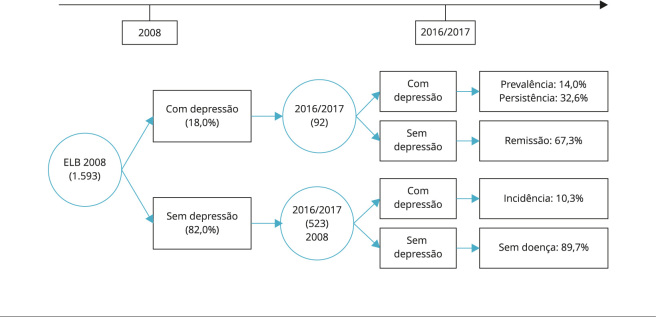
Evolução entre as medidas da *Escala de Depressão Geriátrica* (GDS-15 - *Geriatric Depression Scale*) entre 2008 e 2016/2017. Bagé, Rio Grande do Sul, Brasil.

A análise de incidência cumulativa deste estudo foi restrita a 615 idosos com informações completas na escala GDS-15, tanto na linha de base como no seguimento, que não apresentavam depressão no ano de 2008. Para esses idosos, observou-se a formação de dois grupos de evolução entre as medidas da escala. O primeiro grupo reuniu 523 idosos que apresentaram resultado negativo no rastreamento de sintomas depressivos em 2008, dos quais 54 (10,3%; IC95%: 7,7-12,9) apresentaram resultado positivo no rastreamento de 2016/2017 (casos incidentes). O segundo grupo incluiu os 92 indivíduos que foram rastreados com depressão em 2008, dos quais 30 (32,6%; IC95%: 22,8-42,3) continuaram com sintomatologia depressiva e 62 (67,3%; IC95%: 57,6-77,1) exibiram remissão dos sintomas (Figura 1), observando-se uma incidência cumulativa de depressão de 10,3%.

ELB: estudo de linha de base. Nota: perdas - referem-se aos idosos que não foram localizados durante o acompanhamento, seja por erro no endereço informado em 2008, mudança para outros municípios ou institucionalização.

Na análise ajustada, mantiveram-se associadas ao desfecho as variáveis incapacidade funcional e sair de casa. A incidência de depressão entre idosos com incapacidade para atividades básicas e instrumentais da vida diária foi de 45,1%, com risco 5,18 (IC95%: 3,04-8,81) vezes maior se comparado com idosos que não foram rastreados com incapacidade (8,1%). A incidência de depressão entre os idosos que saíram de casa uma ou nenhuma vez na semana foi de 18,2%, com risco 1,83 (IC95%: 1,11-3,00) vez maior em relação àqueles que saíram de casa duas vezes ou mais por semana no mês que antecedeu a entrevista (7,8%) (Tabela 2).

**Tabela 2:** Análise bruta e ajustada dos fatores associados à incidência de depressão em idosos. Bagé, Rio Grande do Sul, Brasil, 2016/2017 (n = 523).

Variável	Amostra 2016/2017	Incidência de depressão	Análise bruta	Análise ajustada
n (%)	%	RR (IC95%)	RR (IC95%)
Sexo				
Masculino	189 (36,0)	6,9	1,00	1,00
Feminino	334 (64,0)	12,3	1,78 (0,98-3,24)	1,46 (0,81-2,64)
Idade (anos)				
< 75	431 (82,4)	9,0	1,00	1,00
75 ou mais	92 (17,6)	16,3	1,80 (1,03-3,12)	1,44 (0,79-2,64)
Cor				
Branca	425 (81,3)	9,6	1,00	-
Preta/Parda/Amarela/Indígena	98 (18,7)	13,3	1,38 (0,77-2,47)	-
Situação conjugal				
Casado ou com companheiro	299 (57,2)	8,7	1,00	-
Solteiro, separado ou viúvo	224 (42,8)	12,5	1,43 (0,86-2,38)	0,99 (0,56-1,75)
Escolaridade (anos completos de estudo)				
8 ou mais	127 (24,3)	4,7	1,00	1,00
1-7	303 (58,0)	10,5	2,23 (0,95-5,21)	1,15 (0,46-2,86)
Nenhum	93 (17,7)	17,2	3,64 (1,48-8,95)	1,39 (0,51-3,79)
Classificação econômica (ABEP)				
A/B	175 (33,6)	5,1	1,00	1,00
C	191 (36,6)	11,0	2,13 (1,00-4,54)	1,43 (0,64-3,20)
D/E	155 (29,8)	15,5	3,01 (1,44-6,28)	1,54 (0,69-3,45)
Trabalho no último mês				
Sim	93 (17,8)	4,3	1,00	-
Não	430 (82,2)	11,6	2,70 (1,00-7,30)	1,89 (0,73-4,73)
Aposentadoria				
Sim	376 (71,9)	11,1	1,00	-
Não	147 (28,1)	8,1	0,73 (0,39-1,34)	-
Saiu de casa no último mês				
2 vezes por semana ou mais	374 (71,5)	7,8	1,00	1,00
1 ou nenhuma vez por semana	149 (28,5)	18,2	2,33 (1,41-3,85)	1,83 (1,11-3,00)
Tabagismo				
Fumante/Ex-fumante	280 (53,5)	9,2	1,00	-
Não fumante	243 (46,5)	11,5	1,24 (0,74-2,05)	-
Consumo de bebida alcoólica (último mês)				
Não	411 (78,9)	5,4	1,00	-
Sim	110 (21,1)	11,4	2,09 (0,91-4,77)	0,75 (0,30-1,89)
Rede social				
Nível de apoio moderado e forte	334 (63,9)	8,0	1,00	1,00
Nível de apoio fraco	189 (36,1)	14,2	0,76 (1,06-2,92)	1,58 (0,95-2,61)
Número de morbidades com diagnóstico médico				
1	134 (26,0)	7,46	1,00	1,00
2 ou mais	380 (74,0)	11,3	1,51 (0,78-2,93)	1,29 (0,67-2,49)
Incapacidades (AVD e AIVD)				
Sem incapacidade	482 (94,0)	8,1	1,00	1,00
Com incapacidade	31 (6,0)	45,1	5,58 (3,41-9,12)	5,18 (3,04-8,81)

ABEP: Associação Brasileira de Empresas de Pesquisa; AIVD: atividades instrumentais de vida diária; AVD: atividades de vida diária; IC95%: intervalo de 95% de confiança; RR: risco relativo.

Nota: para análise ajustada, empregou-se um modelo hierárquico de quatro níveis de determinação: 1º nível ajustado para sexo, idade, cor da pele, situação conjugal, escolaridade, classificação econômica, trabalho no último mês e aposentadoria; 2º nível ajustado para sair de casa no último mês, tabagismo, consumo de bebida alcoólica, rede social de apoio; 3º nível ajustado para número de morbidades com diagnóstico médico e incapacidades; 4º nível ajustado para incapacidades (AVD e AIVD).

## Discussão

A incidência cumulativa de depressão na coorte de idosos do estudo SIGA-Bagé, no período de oito anos de acompanhamento, foi de 10,3%. No México, com a escala GDS-15, Bojorquez-Chapela et al. ^12^ encontraram incidência de 24% em coorte de idosos de 65-74 anos, acompanhados por um período de 11 meses. Utilizando a mesma escala, na Itália, Yoshida et al. ^30^ encontraram incidência de 16,9% em coorte de idosos com 65 anos ou mais, acompanhados por três anos. Com a escala *Center for Epidemiological Studies - Depression* (CES-D), Batistone et al. ^19^ observaram uma incidência de 15,2% em coorte com idosos de 60 anos ou mais, acompanhados por dois anos, em Juiz de Fora. Collard et al. ^13^, com a escala CES-D, encontraram incidência de 30,6% em coorte de idosos com 65 anos ou mais, acompanhados na Itália por nove anos. A grande variabilidade na incidência de depressão em idosos pode ser atribuída a diferenças metodológicas, com destaque para os instrumentos e tempo de acompanhamento, além dos contextos sociais dos estudos e de heterogeneidade das amostras.

Os resultados relacionados à baixa incidência e à alta taxa de remissão entre quem foi diagnosticado com depressão no ELB reforçam o caráter multidimensional e dinâmico da doença, que alterna episódios curtos e longos ^6^^,^^31^, podendo se tornar recorrente e de curso crônico. Isso destaca sua relevância para a saúde pública e a necessidade de realização de novos estudos longitudinais de base populacional com intervalos menores, com o objetivo de verificar a incidência de depressão em idosos brasileiros. Nesse contexto, é necessário que as políticas públicas implantadas sejam efetivas - fortalecendo relações intersetoriais, como saúde, educação, seguridade social e economia ^32^ - e estejam pautadas na prevenção e promoção da saúde entre os idosos. O Município de Bagé, entre 2008 e 2016/2017, contava com uma rede de atenção e proteção para a população idosa composta por uma Casa de Acolhimento Transitório, uma Casa Dia e um Centro do Idoso. Bagé também tem um Centro de Atenção Psicossocial (CAPS-II) e 86,3% de UBS adotam o modelo da ESF ^33^. Os benefícios desses investimentos podem estar refletidos na alta taxa de remissão e na baixa incidência da depressão identificadas ao longo do seguimento. Wichmann et al. ^34^ destacaram que a participação em grupos de convivência permite que os idosos compartilhem suas angústias, amores, alegrias, afetos, saberes e reduzam sentimentos como medo, tristeza e insegurança. A rede de apoio formada a partir dos grupos de convivência incentiva os idosos a sair de casa, conversar com amigos e/ou profissionais de saúde, receber afeto, trocar experiências e, consequentemente, desenvolver autonomia, melhorar a autoestima e reduzir a vulnerabilidade. O rastreamento de sintomas depressivos entre idosos vinculados a equipes de ESF também permite o desenvolvimento de ações integrais de saúde, criando condições para a promoção de autonomia, integração e participação efetiva do idoso na sociedade ^35^.

A maior restrição no número de vezes que o idoso saiu de casa esteve associada ao risco de depressão após a análise ajustada, evidenciando que os idosos que não saem de casa, ou saem pouco, são mais propensos ao isolamento, à solidão, à baixa autoestima, eventualmente associados a problemas de saúde e incapacidades, contribuindo para a ocorrência de sintomas de depressão ^6^^,^^36^. Estudos desenvolvidos por Harris et al. ^37^ e Bojorquez-Chapela et al. ^12^ relataram que idosos que se sentiam solitários e que não mantinham contato regular com familiares e amigos tinham mais probabilidades de desenvolver depressão, quando comparados àqueles sem essas características.

As incapacidades funcionais para atividades básicas e instrumentais da vida implicam restrições físicas e mentais para os idosos, relacionadas a mobilidade, alimentação, vontade de se vestir e sair de casa para fazer compras ou pagar contas. Essa perda de autonomia afeta a qualidade de vida e pode aumentar o risco de depressão. Nos estudos de Michikawa et al. ^15^, Luppa et al. ^9^ e Stek et al. ^38^, a depressão foi significativamente maior entre os idosos com incapacidade para atividades básicas e instrumentais da vida diária, com risco de 2,64 (IC95%: 1,28-5,46), 2,60 (IC95%: 1,31-5,14) e 2,20 (IC95%: 1,30-3,80), respectivamente.

A avaliação criteriosa da incidência de depressão na população idosa é um dos destaques do estudo. As taxas de incidência e remissão encontradas são representativas da população idosa moradora da zona urbana do Município de Bagé. O instrumento utilizado para rastrear a sintomatologia depressiva em idosos tem alta sensibilidade e especificidade de acordo com os critérios da CID-10 e é recomendado pelo Ministério da Saúde nos *Cadernos de Atenção à Saúde do Idoso*^25^^,^^31^. Os resultados fornecem subsídios para a capacitação dos profissionais de saúde no âmbito da saúde mental e para o desenvolvimento de intervenções pautadas na identificação precoce e na prevenção do agravamento de sintomas depressivos nos idosos.

Uma das limitações do estudo para avaliar a ocorrência de depressão foi o intervalo de oito a nove anos entre as duas medidas. A incidência observada foi, em geral, menor do que a relatada na literatura nacional e internacional que utiliza o mesmo instrumento, não apenas por diferenças demográficas e de contexto social, mas também porque episódios depressivos de curta duração podem ter sido sub-representados. Além disso, o estudo teve 54% de taxa de não respondentes (40% de óbitos, 14% de perdas e recusas), o que reduziu o poder estatístico das análises e afetou a capacidade de identificar associações significativas com depressão de variáveis comumente associadas em outros estudos, como sexo ^9^^,^^12^^,^^19^^,^^39^, idade ^10^, situação conjugal ^12^, nível socioeconômico ^12^ e rede social ^11^^,^^39^. Novos estudos longitudinais de base populacional deverão ser realizados com intervalos menores, com o objetivo de verificar a incidência de depressão em idosos brasileiros e, com isso, subsidiar as políticas de atenção à saúde do idoso.

## Conclusão

A incidência cumulativa de depressão na coorte de idosos do estudo SIGA-Bagé em um período de oito anos de acompanhamento foi de 10,3%. Apresentar incapacidade funcional para as atividades básicas e instrumentais da vida diária e sair de casa uma ou nenhuma vez na semana se associou com maior risco de depressão. Sugere-se o fortalecimento de políticas sociais e de atenção à saúde, em ambientes comunitários seguros com infraestrutura adequada para a população idosa, para a promoção da qualidade de vida e do envelhecimento saudável. Além disso, uma avaliação mais abrangente e qualificada da pessoa idosa na atenção primária à saúde e o incentivo à criação de grupos de convivência permitem o controle da depressão entre os idosos, a preservação da sua saúde física e mental, além de mudanças de hábitos e atitudes que podem diminuir o risco de incapacidade, afastar o idoso da solidão e do isolamento social, promovendo a integração, aumentando a autoestima e melhorando o relacionamento com familiares e amigos ^34^^,^^40^.
